# Exploration of charge states of balanol analogues acting as ATP-competitive inhibitors in kinases

**DOI:** 10.1186/s12859-017-1955-7

**Published:** 2017-12-28

**Authors:** Ari Hardianto, Muhammad Yusuf, Fei Liu, Shoba Ranganathan

**Affiliations:** 1 Department of Molecular Sciences, Macquarie University, Sydney, NSW 2109 Australia; 2 Department of Chemistry, Universitas Padjadjaran, Jatinangor, West Java 45363 Indonesia

**Keywords:** Kinase inhibitors, Ligand charge state, ATP mimic, Molecular modelling, Molecular dynamics simulation

## Abstract

**Background:**

(-)-Balanol is an ATP mimic that inhibits protein kinase C (PKC) isozymes and cAMP-dependent protein kinase (PKA) with limited selectivity. While PKA is a tumour promoter, PKC isozymes act as tumour promoters or suppressors, depending on the cancer type. In particular, PKCε is frequently implicated in cancer promotion, making it a potential target for anticancer drugs. To improve isozyme selectivity of balanol, exhaustive structural and activity relationship (SAR) studies have been performed in the last two decades, but with limited success. More recently, fluorination on balanol has shown improved selectivity for PKCε, although the fluorine effect is not yet clearly understood. Understanding the origin to this fluorine-based selectivity will be valuable for designing better balanol-based ATP mimicking inhibitors. Computational approaches such as molecular dynamics (MD) simulations can decipher the fluorine effect, provided that correct charges have been assigned to a ligand. Balanol analogues have multiple ionisable functional groups and the effect of fluorine substitutions on the exact charge state of each analogue bound to PKA and to PKCε needs to be thoroughly investigated in order to design highly selective inhibitors for therapeutic applications.

**Results:**

We explored the charge states of novel fluorinated balanol analogues using MD simulations. For different potential charge states of these analogues, Molecular Mechanics Generalized Born Surface Area (MMGBSA) binding energy values were computed. This study suggests that balanol and the most potent fluorinated analogue (5*S* fluorine substitution on the azepane ring), have charges on the azepane ring (N1), and the phenolic (C6′′OH) and the carboxylate (C15′′O_2_H) groups on the benzophenone moiety, when bound to PKCε as well as PKA.

**Conclusions:**

To the best our knowledge, this is the first study showing that the phenolate group is charged in balanol and its analogues binding to the ATP site of PKCε. Correct charge assignments of ligands are important to obtain predicted binding energy values from MD simulations that reflect experimental values. Both fluorination and the local enzymatic environment of the ATP site can influence the exact charge states of balanol analogues. Overall, this study is highly valuable for further rational design of potent balanol analogues selective to PKCε.

**Electronic supplementary material:**

The online version of this article (10.1186/s12859-017-1955-7) contains supplementary material, which is available to authorized users.

## Background

**Fig. 1:**
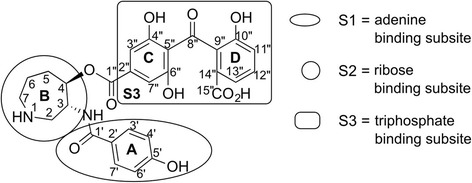
Balanol structure, decomposed into subsites based on structural overlay with ATP

(-)-Balanol (referred to as balanol) is a natural product originally isolated from the fungus *Verticillium balanoides* [1]. It is an ATP mimic [2] as revealed by X-ray crystallographic structures of PKA-bound balanol (1BX6) [3] and ATP (1ATP) [4]. Balanol comprises four ring structures and fully occupies the flexible ATP site (Fig. 1). The benzamide moiety (ring A) occupies the adenine subsite, whereas the azepane moiety (ring B) resides in the ribose subsite. The benzophenone moiety (rings C and D) fills the triphosphate subsite.

(-)-Balanol is a competitive inhibitor of ATP but nonselective for protein kinase A (PKA) and protein kinase C (PKC) isozymes [5]. PKA is known to have tumour promoting activities [6]. Similarly, PKC isozymes regulate gene expression important to the cell cycle, tumorigenesis, and metastatic progression. Most PKC isozymes, however, can act as tumour promoters or suppressors, depending on the type of cancer. For instance, in breast cancer, whereas PKCα, PKCβII, and PKCδ are tumour promoters, PKCβI suppresses the cancer [7]. On the other hand, PKCβI is a promoter and PKCδ is a suppressor in prostate cancer. Of the PKC isoforms, PKCε exhibits clear oncogenic activities and is a potential anti-cancer therapeutic target [7].

Designing inhibitors that are selective to an individual PKC isozyme is very challenging due to the high sequence conservation of ATP sites among PKC isozymes and also other protein kinases, in general [7]. To achieve PKC isozyme selectivity, balanol has been explored intensively in several structure and activity relationship (SAR) studies, with PKA as reference in some of these studies. These SAR studies included modifications to every part of balanol (illustrated in Fig. 1): benzamide (ring A) [8, 9], azepane (ring B) [10], and benzophenone moieties (rings C and D) [11, 12]. Modifications to the benzamide moiety emphasized the important role of C5′OH for PKC inhibition [8]. Derivatization studies on the benzophenone ring, which were replacement of the carboxylic group on ring D with hydrogen, esters, hydroxyl, amide, sulfonamide, or tetrazole, pointed to the importance of the acidic functional group on balanol activity [10, 11]. Some SAR studies were performed by replacing the azepane ring with five-membered pyrrolidine ring but without substantial selectivity improvement [11].

**Fig. 2:**
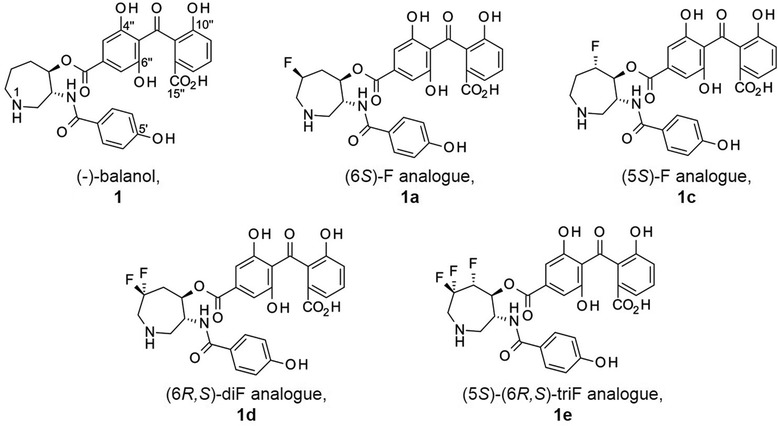
Balanol and its fluorinated analgues **1a**, **1c**, **1d** and **1e**. Fluorine substitutions in the analogues are in the azepane ring, in positions 5 and/or 6 (as labelled in Fig. 1)

More recently, we have successfully introduced stereospecific monofluorination (Table 1: **1a** and **1c**) and multiple fluorine substituents (Table 1: **1d** and **1e**) on the azepane moiety for the first time and measured binding affinities of these fluorinated balanol analogues to PKA and PKC isozymes [13]. Although most of the fluorinated analogues explored in this study (Fig. 2) showed either equal or reduced binding affinity compared to balanol itself (referred to as **1**) across the enzyme panel (Table 1), analogue **1c**, carrying a fluorine substituent at the C5(*S*) position, improves the binding affinity and selectivity to PKCε. The introduction of a small fluorine substituent on the azepane moiety does not introduce significant size change to balanol [14, 15]. However, fluorine can provide complex physicochemical perturbation and stereoelectronic effects that alter the overall confirmation of balanol for selective binding amongst highly homologous ATP sites of protein kinases [13].

**Table 1:** Table 1 Dissociation constant (*K*_d_) and binding affinity values of balanol analogues to PKA or PKCε

Protein kinase	(-)-balanol, **1**	(6*S*)-F analogue, **1a**	(5*S*)-F analogue, **1c**	(6*R,* *S*)-diF analogue, **1d**	(5*S*)-(6*R,S*)-triF analogue, **1e**
	*K*_d_ (nM)				
PKA	5.9 ± 0.5	7.9 ± 0.5	6.4 ± 0.1	9.2 ± 0.8	43 ± 4
PKCε	0.73 ± 0.06	19 ± 8	0.4 ± 0.02	110 ± 19	38 ± 9.5
	$$ \Delta {G}_{experiment}^{{}^{\circ}} $$ (kcal.mol^−1^)				
PKA	−11.30 ± 0.05	−11.12 ± 0.03	−11.25 ± 0.01	−11.03 ± 0.05	−10.11 ± 0.05
PKCε	−12.54 ± 0.05	−10.60 ± 0.21	−12.90 ± 0.03	−9.55 ± 0.09	−10.19 ± 0.14

Understanding the fluorine effect on the binding of balanol analogues to PKA as well as PKCε is an important aspect of further development of balanol-based inhibitors. A rapid way to acquire this understanding is by computational approaches. Our earlier computational investigation using a molecular docking approach showed that, compared to unsubstituted balanol, additional protein-ligand interactions in the ATP site can be conferred by fluorination [13]. In addition to molecular docking, molecular dynamics (MD) simulations can provide a deeper understanding of the binding of fluorinated balanol analogues to PKA as well as to PKCε. An MD simulation allows the investigation of intermolecular interaction dynamics between the ligand and residues at the binding site [16]. It also provides insight into the conformational space explored by the ligand during binding as well as the binding energy from ensemble conformations. Furthermore, since protein kinases are flexible enzymes capable of induce-fit interactions [2], MD simulations provide an opportunity to investigate the effect of this plasticity in ligand binding [17] to PKC and PKA.

In MD simulations, the charge state of each ligand needs to be specified *a priori* [18]. Balanol has several polar protic functional groups in its structure (Fig. 1) [1], comprising a secondary amine on the azepane ring (ring B), a carboxylic acid group on the benzophenone ring (ring D), and four phenolic groups on the benzamide (ring A) and the benzophenone (rings C and D) moieties. These basic and acidic functional groups potentially adopt multiple charge states depending on the pH of the ATP-binding site. Charge state variation of balanol were computationally investigated in a previous study [19]. The study suggested that charges on the carboxylic (ring D) and the amine (ring A) would be necessary for balanol binding to the ATP site of PKA, while the negative charges on phenolic groups were considered to have only a small contribution to binding affinity and were therefore considered neutral.

The charge states of fluorinated balanol analogues may differ from that of the parent compound, balanol. The introduction of a highly electronegative fluorine substituent may change the charge state profile of balanol analogues. Hence, an investigation of the charge states of fluorinated balanol analogues will yield highly valuable information in order to assess which of these charge states act as the major contributing species for kinase binding at biological assay pH (7.40) [13]. Similar to the earlier charge state investigation of balanol [19], we calculated the p*K*_a_ value [20] for each functional group of the balanol analogues, in order to identify specific charge states relevant to binding. Each analogue, with its respective charge state bound to either PKA or PKCε, was subjected to an MD simulation (as detailed in the Methods section). Subsequently, binding energy values for each protein-ligand pair were determined using a Molecular Mechanics Generalized Born Surface Area (MMGBSA) approach (described in Methods). The results show that the fluorine substituent(s) and the local environment of the ATP site together determine the charge states of bound fluorinated balanol analogues under biological assay pH. In the ATP site of PKCε, balanol **1** and the most promising fluorinated analogue **1c** have charges on N1, C6′′OH, and C15′′O_2_H, whereas **1d** and **1e** bear charges only on C6′′OH and C15′′O_2_H. These balanol analogues also retain their charge states when they are bound to PKA. In contrast, analogue **1a**, which is fluorinated on C6(*S*), exhibits different charge states in the ATP sites of PKA and PKCε. Specifically, N1 and C6′′OH of 1a are neutral in PKA, whereas PKCε-bound **1a** may exist as two species: one bearing ionised N1 and C6′′OH and the other possessing both functional groups in the uncharged state. The information obtained from this study will be beneficial for further rational design of balanol analogues to improve binding affinity for PKCε, as well as improved PKC isozyme selectivity.

## Methods

### Initial p*K*_a_ assignment

p*K*_a_ values of basic and acidic functional groups present in all analogues were estimated using Marvin Suite 17.1.30.0 [20, 21], at physiological pH (7.4). The predicted p*K*_a_ values were used as the starting point for assigning charge to the basic and acidic groups.

### Homology modelling

The catalytic domain of human PKA (UniProt ID: P17612) and PKCε (UniProt ID: Q02156) binding balanol were prepared using a homology modelling approach as reported previously [13]. We used a crystal structure of mouse PKA-balanol (PDB ID: 1BX6) [3] as the template for homology modelling of human PKA. For human PKCε, the homology model was built based on two crystal structure templates: from mouse PKA and human PKCη (PDB ID: 3TXO). The templates were structurally aligned using the jCE algorithm [22] to examine the equivalency of both structures. Subsequently, the sequence of human PKCε was aligned to 1BX6 and 3TXO sequences using CLUSTALX 2.1 [23]. In addition, we manually edited the sequence alignment to map the ‘open’ conformation [24] at the Gly-rich loop (GXGXXG) from 1BX6 to the PKCε model. The sequence homology and identity of human PKA and PKCε to the template sequences were calculated using EMBOSS Needle [25].

The homology modelling was performed in the presence of balanol, from mouse PKA-balanol (PDB ID: 1BX6) to retain the three dimensional (3D) characteristics of the ATP binding site of the resulting homology model. The 3D models of the protein kinases with bound balanol was built using MODELLER 9.14 [26]. The resulting models were ranked using the Discrete Optimised Protein Energy (DOPE) score [27]. The score represents the quality of the model and the lower the score, the more native-like the corresponding model. Additionally, we overlaid models from several Modeller runs and selected the model which has residues adopting consensus conformations. The model was further assessed using the Ramachandran plot from the PROCHECK webserver [28]. Molecular surface electrostatic potentials (MSEPs) of PKA and PKCε were calculated using Adaptive Poisson-Boltzmann Solver (APBS) Version 0.5.1 [29] as implemented in AutoDockTools Version 1.5.6 [30].

### Molecular dynamics simulation preparation and protocol

Fully activated human PKA and PKCε have phosphorylated residues at several specific sites [31]. Thus, we added a phosphoryl moiety on PKA to each of the phosphorylated residues: Thr198 and Ser339 using Discovery Studio Visualizer [32]. Likewise, phosphoryl moieties were also appended on Thr566, Thr710, and Ser729 of PKCε. The initial poses of bound balanol and its fluorinated analogues were adopted from the conformation of the ligand in 1BX6, where mouse PKA was co-crystallised with balanol.

Atomic charges and force-field parameters are needed to describe interatomic interactions in MD simulations. For balanol and its analogues in various protonation states, their atomic charges were calculated using the Austin model 1 - bond charge corrections (AM1-BCC) [33] procedure using Amber tools [34]. Their parameters were assigned from the General Amber Force-fields (GAFF) [35] and those missing in GAFF were determined using the *parmchk* program in Amber tools. Proteins and their side chains were assigned Amber ff14SB [36] force-field parameters, whereas phosphorylated amino acids used phosaa10 [37] parameters.

Before MD simulation, each complex of either PKA- or PKCε-balanol-analogue was solvated in an explicit TIP3P water model using the LEaP module in Amber tools. The minimum buffering distance between the protein and box boundary was set to 10 Å. A number of Na^+^ and Cl^−^ ions were then added to obtain neutral charge and a concentration of 0.15 M, which is the equivalent of salt concentration under physiological conditions.

The MD simulation protocol was adapted from Homeyer and Gohlke [38]. For each simulation, an energy minimisation with high restraint (25 kcal.mol^−1^ Å^−2^) to the solute was performed, followed by one with low restraint (5 kcal.mol^−1^ Å^−2^). After minimisation, the system temperature was increased to 300 K under NVT condition over 50 ps, followed by equilibration as described in next sentences. In the next 50 ps of NPT simulation, the system density was adjusted to 1 g.cm^−1^. Subsequently, restraint on solute was gradually decreased by 1 kcal mol^−1^ Å^−2^ every 50 ps over the rest of the NVT simulation, with the last 50 ps simulation carried out without restraint.

Production-phase simulations of 100 ns were conducted at 300 K under the NPT ensemble conditions for all systems, where the periodical boundary conditions were applied. During simulation, long-range electrostatic interactions were treated by particle-mesh Ewald (PME) method. For short-range non-bonded interactions, we employed a 10 Å cut-off which was also implemented previously by Fisette et al. [39] and Kumar et al. [40]. All bonds involving hydrogen atoms were constrained by using the SHAKE algorithm [41]. Algorithms of Berendsen barostat [42] and Langevin thermostat [43] were used to maintain constant pressure and temperature, respectively. All production-phase simulations were run using GPU accelerated particle-mesh Ewald molecular dynamics (PMEMD) as implemented in Amber16 [34].

### Binding energy calculation

Experimental binding energy or binding affinity values between balanol analogues and PKA or PKCε were derived from the dissociation constant (*K*_d_) values as presented in Table 1 [13]. At equilibrium and under standard conditions, the binding energy is related directly to the equilibrium constants and, thus, can be calculated using formulae as follows:

1 $$ \Delta  {G}_{experiment}^{{}^{\circ}}=- RTln\left({K}_a\right)= RTln\left({K}_d\right) $$

where $\Delta  {G}_{experiment}^{{}^{\circ}}$ is the experimental binding energy, *K*_a_ and *K*_d_ are the association and dissociation constants, respectively, R is the universal gas constant, and T is the absolute temperature.

Calculated binding energy values between balanol analogues and PKA or PKCε were estimated using Molecular Mechanics Generalised Born Surface Area (MMGBSA) approach [44] as implemented in MMPBSA.py [45]. The MMGBSA binding free energy ($\Delta  {G}_{MMGBSA}^{{}^{\circ}}$) is calculated by:

2 $$ \Delta  {G}_{MMGBSA}^{{}^{\circ}}={\left\langle {G}_{complex}\right\rangle}_i-{\left\langle {G}_{enzyme}\right\rangle}_i-{\left\langle {G}_{ligand}\right\rangle}_i $$

Here 〈*G*_*x*_〉_*i*_, with *x* is complex, enzyme, or ligand, denotes the average value of the MMGBSA free energy for complex, enzyme, or ligand over snapshots *i* obtained from MD trajectories. *G*_*x*_ can be broken down into three components:

3 $$ {G}_x={E}_{MM}+{G}_{solv}^{GB}+{G}_{solv}^{SA} $$

where *E*_*MM*_ is the gas phase energy, ${G}_{solv}^{GB}$ the electrostatic portion of solvation energy determined using Generalised Born (GB) implicit solvent model, and ${G}_{solv}^{SA}$ the hydrophobic contribution to the solvation energy. The hydrophobic contribution is approximated by the Linear Combination of Pairwise Overlaps (LCPO) method [46]. *E*_*MM*_ is estimated by the molecular mechanics energy of the molecule consisting bond, angle, and torsion energies as well as van der Waals and electrostatic interaction which can be expressed as:

4 $$ {E}_{MM}=\sum \limits_{bond s}{E}_{bond}+\sum \limits_{angle s}{E}_{angle}+\sum \limits_{torsion s}{E}_{torsion}+\sum \limits_{i\ne j}^{atoms}{E}_{vdW}+\sum \limits_{i\ne j}^{atoms}{E}_{electrostatic} $$

*E*_*bond*_, *E*_*angle*_, and *E*_*torsion*_terms were eliminated in the computation, since only protein-ligand complexes were subjected to MD simulations (single trajectory) [18]. $\Delta  {G}_{MMGBSA}^{{}^{\circ}}$ for each balanol analogue bound to PKA or PKCε was calculated within a 10-ns-sliding window every 10 ns, 100 snapshots per sliding window. Plots of $\Delta  {G}_{MMGBSA}^{{}^{\circ}}$ values and their correlation coefficient with experimental binding energy were visualised using a ggplot2 package [47] in R statistical software [48].

## Results

### The effect of fluorine incorporation on acidity and basicity of functional groups on balanol analogues

As depicted in Fig. 1 and Table 2, balanol has polar protic functional groups comprising one basic and five acidic groups. The basic functional group is a secondary amine (N1) on the azepane moiety (ring B). The acidic ones consist of a carboxylic acid (C15′′O_2_H) on ring D of benzophenone and four phenolic groups: one (C5′OH) on the benzamide moiety; two (C4′′OH and C6′′OH) on ring C and one (C10′′OH) on ring D of benzophenone.

**Table 2:** Predicted p*K*_a_ values of the ionisable functional groups on (-)-balanol and its analogues

Analogue	Marvin estimates of p*K*_a_					
	N1	C5′OH	C4′′OH	C6′′OH	C10′OH	C15′′O_2_H
**1**	9.65	8.58	7.94	6.52	7.22	2.98
**1a**	8.22	8.74	7.77	6.51	7.18	2.98
**1c**	9.37	8.55	7.93	6.52	7.22	2.98
**1d**	6.20	8.62	7.96	6.73	7.28	2.98
**1e**	6.20	8.62	7.96	6.73	7.28	2.98

To get a preliminary estimate before performing charge state exploration on balanol and its fluorinated analogues (shown in Fig. 2), we calculated p*K*_a_ values of these polar protic functional groups (Table 2) using Marvin [20, 21]. Marvin predicts p*K*_a_ values mainly based on calculated empirical partial charges and parameterized hydrogen bonds, and has also been utilized by Drugbank [49] for p*K*_a_ prediction. For the natural balanol (labelled **1** in Fig. 2), Marvin predicted that the amine N1 on the azepane ring has a p*K*_a_ value of 9.65 and the phenolic C5′OH on the benzamide moiety has a p*K*_a_ value of 8.58. On the benzophenone moiety, the predicted p*K*_a_ value for the carboxyl C15′′O_2_H is 2.98. The phenolic C4′′OH and C6′′OH on the ring C have p*K*_a_ values of 7.94 and 6.52, respectively, whereas that of C10′′OH on the D ring is 7.22. Hence, the natural balanol **1** has a positive charge on N1 and negative charges only on C6′′OH and C15′′O_2_H at biological assay pH (7.40). According to Marvin, the introduction of fluorine on the azepane ring, as seen in all of the fluorinated balanol analogues, affects only very slightly the p*K*_a_ of C5′OH on the benzamide moiety. The predicted p*K*_a_ values of the benzamide phenolic moiety for all analogues are above 8.00, suggesting that this moiety is uncharged at biological assay pH (7.40). Uncharged benzamide may benefit the binding of balanol analogues to kinases, particularly in the adenine subsite. The adenine subsites of both PKA and PKC are dominated by hydrophobic residues that favour uncharged moieties or ligands.

On the azepane ring itself, the introduction of fluorine influences the basicity of the amine group (N1). The presence of a highly electronegative fluorine atom on C6(*S*) (analogue **1a**) decreases the basicity of the N1, as indicated by its predicted p*K*_a_ (8.22) [50]. This electron withdrawing effect is less pronounced for 1c where the fluorine atom (on C5(*S*)) is further away from N1. As shown by Marvin prediction, p*K*_a_ of N1 on **1c** is only slightly decreased to 9.37. Meanwhile, di- and trifluorination on C6(*R,S*) (**1d**) and C5(*S*)-C6(*R,S*) (**1e**), respectively, are predicted to dramatically lower the basicity of the N1 as shown in Table 2. Based on these predicted p*K*_a_ values, the N1 groups on **1**, **1a**, and **1c** are expected to be protonated and have a positive charge at biological assay pH (7.40), whereas the N1 groups on **1d** and **1e** are unprotonated (uncharged).

Predicted p*K*_a_ values suggest that the presence of fluorine on the azepene ring slightly alters the acidity of polar protic functional groups on the benzophenone moiety. These acidity alterations do not affect the charge states of functional groups on the benzophenone moiety. Marvin predicts that the all balanol analogues have negative charges on C6′′OH and C15′′O_2_H, while C4′′OH and C10′OH are neutral at the biological assay pH (7.40).

Wong et al. [19] reported only a negative charge on the benzophenone carboxylate (C15′′O_2_H) and a positive charge on the amine (N1) azepane of balanol, when bound to the ATP site of PKA. Marvin results additionally suggest a negative charge on C6′′OH.

### Charge state exploration of balanol and its fluorinated analogues in the ATP site of PKA

We first assigned the charge state for balanol analogues as reported previously [19], keeping the phenolic groups uncharged. The charge on C15′′O_2_H and N1 are known to be useful for binding of balanol to the ATP site of PKA. Thus, we assigned C15′′O_2_^−^ and N1H_2_^+^ for **1**, **1a**, and **1c** (Table 3, column B). For **1d** and **1e** (Table 3, column B), their amine groups (N1) were kept uncharged since their predicted p*K*_a_ values (Table 2) are well below the pH of biological assays (7.40). All these analogues with respective charge states were labelled as a charge state combination **I** (Table 4). The balanol moiety in the PKA model was then replaced with the analogue in the correct charge state, neutralized with counter ions (Na^+^ or Cl^−^), and solvated by explicit TIP3P water model. Additional Na^+^ or Cl^−^ ions were also added to reach the physiological salt concentration of 0.15 M. Every solvated complex of PKA-bound balanol analogue was subsequently subjected to MD simulation to yield a trajectory of 100 ns. From each trajectory of PKA-bound balanol analogues, binding energy values were calculated within a 10-ns sliding window every 10 ns using MMGBSA approach.

**Table 3:** Charge states of balanol analogues

Analogue	Charge state selected, indicated by x			
	A	B	C	D
	COO^−^; NH; OH	COO^−^; NH_2_^+^; OH	COO^−^; NH_2_^+^; O^−^	COO^−^; NH; O^−^
**1**		×	×	
**1a**	×	×	×	
**1c**		×	×	
**1d**	×			×
**1e**	×			×

*COO* ^-^ Carboxyl (C15′′O_2_^−^), *NH* Amine (N1H), *NH*_*2*_^*+*^ Ammonium (N1H_2_^+^), *OH* Phenol (C6′′OH), *O* ^-^ Phenolate

**Table 4:** Combination of charge states

Analogue	Charge state^a^ combination		
	**I**	**II**	**III**
**1**	B	C	C
**1a**	B	C	A
**1c**	B	C	C
**1d**	A	D	D
**1e**	A	D	D

^a^A, B, C and D as defined in Table 3

$\Delta  {G}_{MMGBSA}^{{}^{\circ}}$ profiles of PKA-bound balanol analogues (Additional file 1: Figure S1) show that the calculated binding energy values do not corroborate well with experiment results (Table 1). The experimental binding affinity values in Table 1 suggest that **1**, **1a**, **1c**, and **1d** bind to PKA with comparable affinities, whereas **1e** is a significantly weaker binding partner. Similarity in calculated $\Delta  {G}_{MMGBSA}^{{}^{\circ}}$ values, however, is observed for **1a** and **1e**, but not for **1**, **1a**, **1c**, and **1d** (Additional file 1: Figure S1). In addition, a good correlation coefficient (*r* ^2^) between the experimental and calculated binding energy only appears once in the first 10-ns of trajectory, after which the *r* ^2^ values fall to below 0.50 until the end of the simulation. Clearly, we need to explore charge states other than those reported by Wong et al. [19].

According to the Marvin prediction (Table 2), the phenolic group at C6′′ among all analogues has p*K*_a_ values ranging from 6.50 to 6.75. Thus, at the pH of biological assays (7.40), this functional group is most likely to bear a negative charge. The phenolate group (C6′′O^−^) was then assigned for **1**, **1a**, **1c** (Table 3, column C), and for **1d**, and **1e** (Table 3, column D) and grouped as charge state combination **II** (Table 4). All analogues, in the PKA-bound state, were then subjected to MD simulations to yield trajectories of 100 ns. Extracted from MD trajectories, the *r* ^2^ profile shows that $\Delta  {G}_{MMGBSA}^{{}^{\circ}}$ values of all PKCε-bound analogues still show poor correlation with experimental data (Additional file 2: Figure S2B). Most of the *r* ^2^ values are around 0.10.

**Fig. 3:**
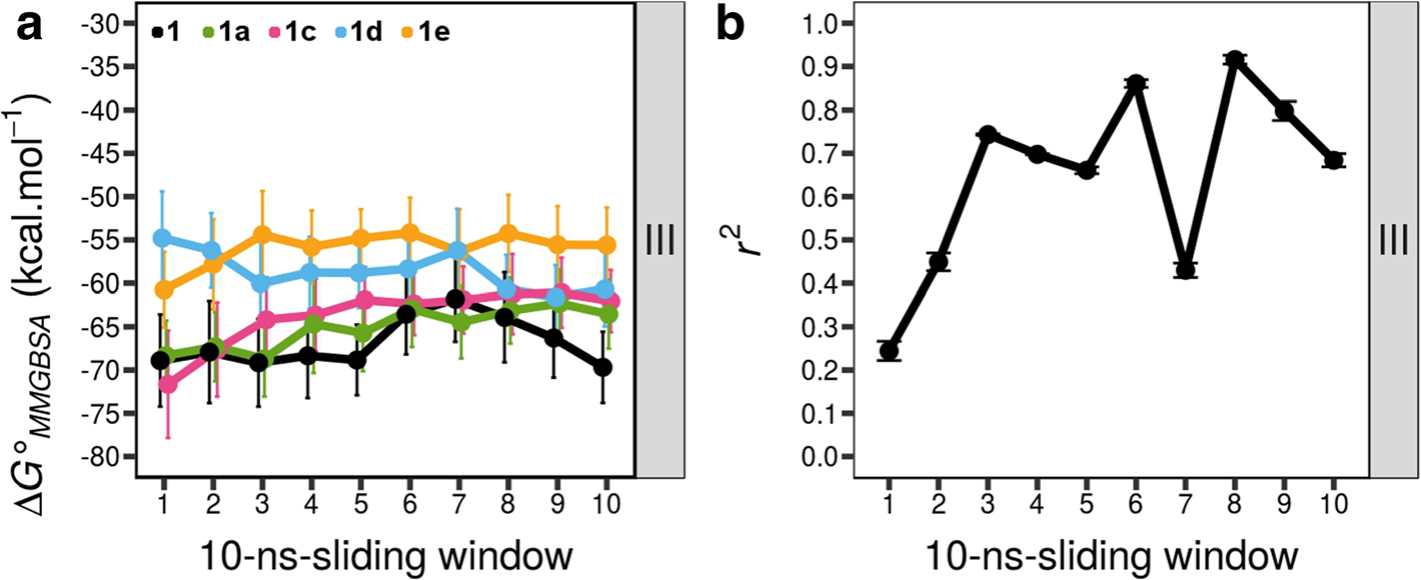
**a** Trajectory of $\Delta  {G}_{MMGBSA}^{{}^{\circ}}$ of PKA-bound balanol analogues in charge state combination **III** and **b** the correlation coefficients to experimental binding energy over 100 ns of MD simulations. Each data point in **a** was obtained from a 10-ns sliding window every 10 ns. Error bars in **a** were obtained from $\Delta  {G}_{MMGBSA}^{{}^{\circ}}$ calculations of 100 snapshots within 10 ns trajectory. Error bars in **b** were derived from the errors of experimental *K*_d_ values

From the $\Delta  {G}_{MMGBSA}^{{}^{\circ}}$ profile of PKA-bound balanol analogues in the charge state combination ** II** (Table 4), we noticed that the calculated binding energy of **1a** is an outlier (Additional file 2: Figure S2A). In analogue **1a**, the fluorine atom is on C6(*S*) which is in the *β*-position from N1 that has a p*K*_*a*_ value of 8.22. Given that Marvin has a root-mean-squared (RMS) error of about 1 p*K*_*a*_ unit [21], this value is in the range 7.22–9.22, making this amine either uncharged or positively charged at pH 7.4. Hence, we explored another charge state for **1a** in which N1 is neutral. The C6′′OH was also treated as uncharged for **1a** as reported before [19]. We grouped this new charge of **1a** in combination **III** with the remaining analogues retaining their charge states in combination **II** (Table 3). Subsequently, we run another set of MD simulations. The result show that the $\Delta  {G}_{MMGBSA}^{{}^{\circ}}$ profile of **1a** (Fig. 3a) moves closer to that **1**, **1c**, and **1d**. As expected, *r* ^2^ profiles for PKA-bound analogues were improved when we use charge state combination **III** (Fig. 3b). This combination has average *r* ^2^ of 0.72, calculated from a trajectory between 40 and 100 ns. These explorations suggest that balanol analogues most likely have charge states as listed in combination **III** when bound to the ATP site of PKA.

### Charge state exploration of balanol and its fluorinated analogues in the ATP site of PKCε

In the charge state exploration of balanol analogues in the ATP site of PKCε, we used the same approach as described above for PKA (Table 4). Using charge state combination **I**, however, resulted in $\Delta  {G}_{MMGBSA}^{{}^{\circ}}$ profiles for PKCε-bound balanol analogues (Additional file 3: Figure S3A) that disagree with the experimental results, where the order of binding affinity is **1c** > **1** > > **1a** > **1e** > > **1d**. The analogues having the strongest experimental binding affinity for PKCε, **1** and **1c**, also have the lowest  calculated binding energy values for charge state **I**. Moreover, throughout the trajectory, all *r* ^2^ values are below 0.50 (Additional file 3: Figure S3B).

**Fig. 4:**
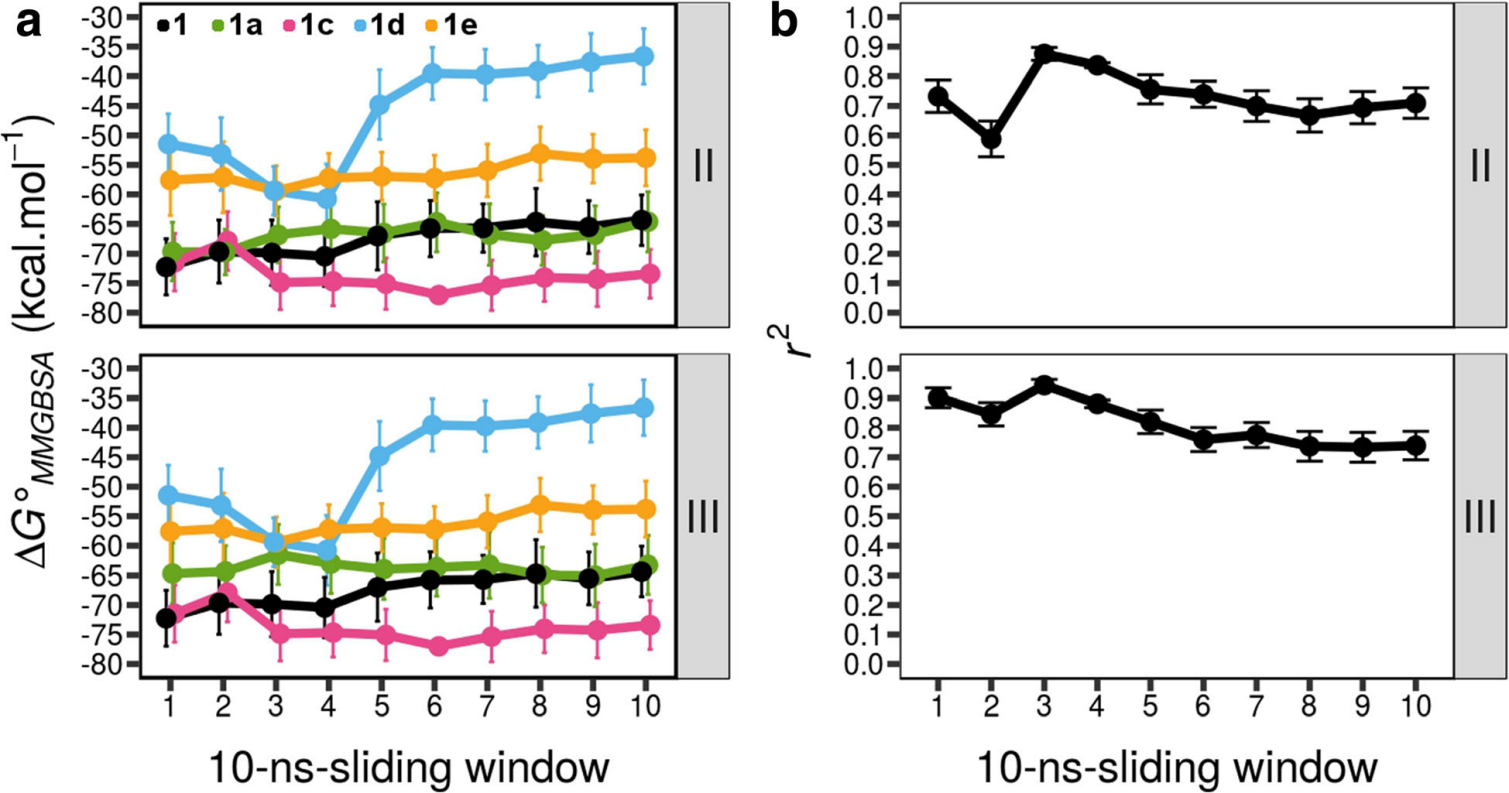
**a** Trajectory of $\Delta  {G}_{MMGBSA}^{{}^{\circ}}$ of PKCε-bound balanol analogues in charge state combinations **II** and **III** and **b** the respective correlation coefficients to experimental binding energy over 100 ns of MD simulations. Row labels (**II**, and **III**) indicate charge state combinations. Each data point in **a** was obtained from a 10-ns sliding window every 10 ns. Error bars in **a** were obtained from $\Delta  {G}_{MMGBSA}^{{}^{\circ}}$ calculations of 100 snapshots within 10 ns trajectory. Error bars in **b** were derived from the errors of experimental *K*_d_ values

Combination **II** gave $\Delta  {G}_{MMGBSA}^{{}^{\circ}}$ profiles that follow the experiment result, where **1c** and **1d** are the strongest and weakest ligands, respectively, among other analogues (Fig. 4a combination **II**). The *r* ^2^ profile also exhibits good correlations between $\Delta  {G}_{MMGBSA}^{{}^{\circ}}$ and experimental binding energy values (Fig. 4b combination **II**). Average *r* ^2^ for combination **II** is 0.73, which was calculated between 40 and 100 ns of trajectory.

Interestingly, charge state combination **III** yielded a similar $\Delta  {G}_{MMGBSA}^{{}^{\circ}}$ profile to that using combination **II** (Fig. 4a combination **III**). Furthermore, the *r* ^2^ profile shows slightly improved correlations (Fig. 4b combination **III**) with average of 0.78.

These results suggests that PKA-bound balanol analogues most likely possess the same charge states to those bound to PKCε, except for **1a**. Balanol **1** and the most promising fluorinated analogue (**1c**) bear charges on the azepane amine, N1 and on the phenol C6′′OH and the carboxyl C15′′O_2_H, of the benzophenone moiety. Analogues **1d** and **1e** retain the carboxylate and phenol group charges while the amine group remains uncharged. While PKA-bound **1a** has uncharged N1 and C6′′OH, **1a** may adopt two charge states when it is bound to the ATP site of PKCε, with N1 being either charged or neutral.

## Discussion

### The influence of fluorine substituent and local environment of ATP site on charge state of balanol analogues

Fluorine is a highly electronegative substituent with very little steric effect on the the balanol molecule [14, 15]. The inductive effect of fluorine substitution on the azepane ring alters the acidity and basicity of polar protic functional groups in the balanol system. This in turn influences the charge states of balanol analogues and consequently their binding to the ATP site of protein kinases such as PKA and PKCε. We find that fluorination can significantly affect the charge state of N1 at bioassay pH (7.40). For analogue **1a**, monofluorination on the carbon *β* to N1 can reduce the basicity of the amine group to leave it unprotonated at the bioassay pH. Installation of a single fluorine on the carbon *γ* to N1 (**1c**) also reduces the basicity of N1, but it does not change the charge state at the bioassay pH. A more pronounced effect of charge state alteration is found by our calculation when di- or trifluorination is introduced on the azepane ring.

**Fig. 5:**
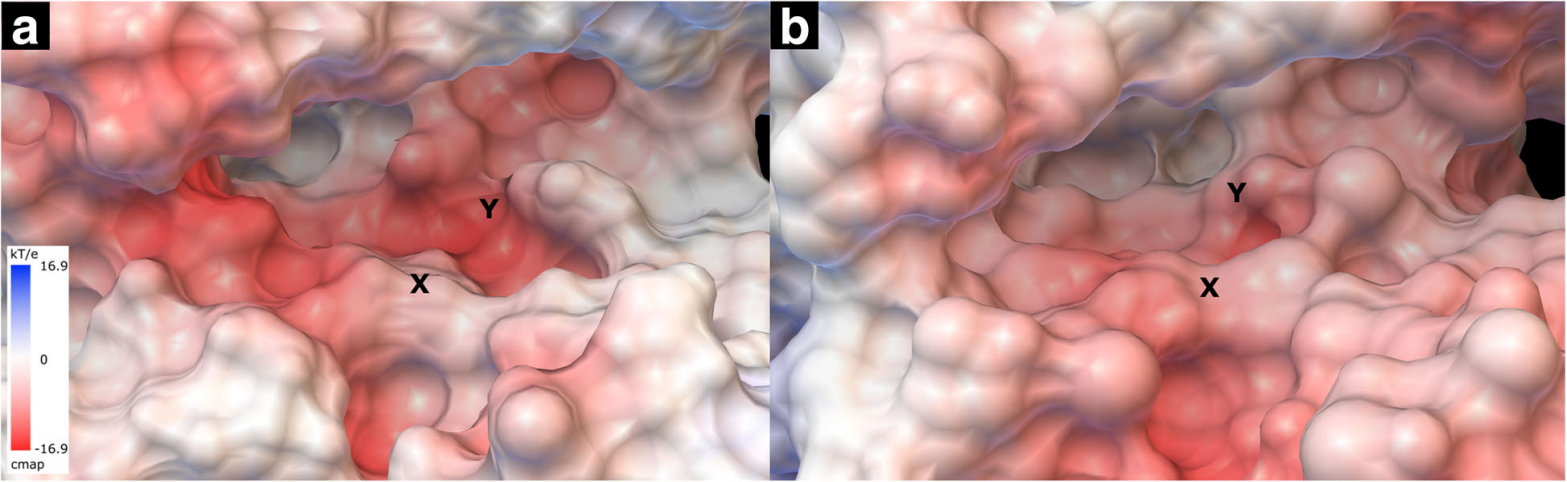
Molecular surface electrostatic potential (MSEP) of ATP sites of **a** PKA and **b** PKCε. X indicates the position of N1, whereas Y is the site for C6′′OH

Our MD simulations suggest that PKA-bound balanol analogues in charge state combination **III** gives $\Delta  {G}_{MMGBSA}^{{}^{\circ}}$ values well correlated with experiment results. In this combination list, **1a** has an uncharged N1. Meanwhile, for balanol analogues bound to the ATP site of PKCε, good agreements between simulated $\Delta  {G}_{MMGBSA}^{{}^{\circ}}$values and experiment results are observed for charge state combination **II** and **III**. These results suggest that **1a** possesses N1 either in an uncharge or charge state when it is bound to the ATP site of PKCε. The charge states of balanol in the bound form may also be influenced by the local environment of each subsite in the ATP site. The ribose subsite in PKA where azepane resides has a molecular surface electrostatic potential (MSEP) that is close to neutral (Fig. 5a indicated by X). This may retain the N1 of azepane ring uncharged. For PKCε, its MSEP at the ribose subsite indicates a slight negative or acidic environment (Fig. 5b indicated by X) which allows **1a** to be protonated at N1, whereas the others are uncharged or unprotonated at N1. This may in part explain why analogue **1a** has comparable binding affinity to that of **1 **in the PKA ATP pocket, as the protein active site is neutral and less sensitive to the charge state of the ligand at N1. For the ATP pocket of PKCε that is more acidic and negatively charged, the neutral charge state of N1 of **1a** would cause significant loss in ionic interactions, which is consistent with the experimental observation that **1a** is a significantly worse ligand compared to  native balanol (**1**). A positive charge on N1 of the azepane ring may be an indicator for balanol analogues to strongly bind the ATP site of PKCε, as reflected by **1c**, the best ligand among the analogues in this study.

In addition to N1, our charge state exploration also suggests that C6′′OH on **1a** binds differently in the ATP site of PKA from that of PKCε. In the ATP site of PKA, the C6′′OH is in its uncharged state, whereas when **1a** is bound to PKCε, it may exist in either the uncharged or the charged state. The nature of the triphosphate subsites between PKA and PKCε differ, which may influence the charge state of C6′′OH in the case of **1a**. PKCε possesses a less negative or less acidic MSEP triphosphate subsite (indicated by Y in Fig. 5) than PKA.

### Molecular dynamics binding of balanol analogues

Balanol analogues exhibit dynamics $\Delta  {G}_{MMGBSA}^{{}^{\circ}}$ profiles over the MD simulation period (see Fig. 4a: charge combination **III**). The most striking feature is observed on the $\Delta  {G}_{MMGBSA}^{{}^{\circ}}$ profile of PKCε-bound **1d** (in cyan line). The analogue **1d**, as the weakest binder to PKCε, shows binding affinity increments for the first 40 ns of MD simulation to around −60 kcal.mol^−1^, but its binding affinity dramatically decreases to −40 kcal.mol^−1^ afterwards and remains stable until the end of simulation. This may indicate that even though the binding affinity between **1d** and the ATP site of PKCε initially increases, interactions of both ligand and receptor in the entropic term may be unfavorable for the whole PKCε system. As a result, the binding affinity decreases or becomes more positive (Fig. 4a charge combination **III** of PKCε-bound **1d**). This transient higher binding affinity can be misleading, if the binding affinity is not thoroughly simulated as a profile of time-sliding window.

An interesting event is also observed on the $\Delta  {G}_{MMGBSA}^{{}^{\circ}}$ profile of PKCε-bound **1c**. Being the analogue with highest affinity to PKCε, the binding affinity of **1c** decreases to −68 kcal.mol^−1^ for the first 20 ns, but then dips to and remains stable at around −75 kcal.mol^−1^. This may suggest that, during the simulation, **1c** can alter its conformation to adapt to the flexible ATP site of PKCε. This conformation change in the ATP site may incur an entropic cost that is paid off by enthalpic compensation through induced fit. Such entropy-enthalpy compensation seems to benefit the binding affinity of **1c** to PKCε.

### The effect of ligand charge states on predicted binding energy values

Through this study, we demonstrated the importance of assigning correct charge states to ligands in binding energy calculations, which also has been reported previously [18]. Improper charge state assignments to ligands lead to disagreements between calculated and experimental binding energy values. For instance, we observed such disagreements on binding energy values of charge state combination **I** for both PKA- and PKCε-bound balanol analogues (Additional file 1: Figure S1 and Additional file 3: Figure S3, respectively). Assigning this combination to both PKA- and PKCε-bound balanol analogues yields poor correlation coefficients between calculated and experimental binding energy values. The correlations for PKA-bound balanol analogues are improved when combination **III** is employed (Fig. 3), whereas for PKCε-bound balanol analogues, satisfactory correlations are obtained by assigning combinations **II** for all analogues excepting **1a**, for which combination **II** or **III** is acceptable (Fig. 4).

Charge distributions of the two ATP sites, which are represented by MSEPs, can have different abilities to accommodate charge interactions of ligands. Meanwhile, fluorination allows charge state diversification of ligands. Altogether, the charge distribution of ATP sites and ligand fluorination could be utilized as a tool for conferring specificity constraints of ligands.

## Conclusions

(-)-Balanol has several polar protic functional groups that include basic and acidic groups. These groups can have different charge states depending on the environmental pH. Furthermore, the presence of fluorine atom in the azepane ring alters the acidity and basicity of functional groups in the balanol system. This alteration can change the charge states of major binding species of balanol analogues at biological assay pH. In addition, the charge states of balanol analogues can be influenced by the local environment of the ATP site.

Here, we investigated charge states of novel fluorinated balanol analogues. This investigation suggests that PKCε-bound balanol **1** and its fluorinated analogues **1c**, **1d** and **1e** adopt the same charge states when bound to PKA, while **1a** alone shows different charge states. For the first time and to the best our knowledge, we show that in addition to charges on N1 and C15′′O_2_H, a charged phenolate group (C6′′O^−^) is also important for the binding of balanol analogues in the ATP site of protein kinases. This is supported by the evaluation of MMGBSA binding energy values that correlate well with experimental data.

We also found that the charge state of the ligand can influence the calculated binding energy values, as previously observed [18, 19]. Correct forms of charge states are important to generate reasonable binding energy from MD simulations. Ultimately, the insight from this study will help guide further rational designs of balanol analogues for improved selectivity of PKC isozymes and other related kinases.

## Additional files

Additional file 1: Figure S1. A. Trajectory of $\Delta  {G}_{MMGBSA}^{{}^{\circ}}$ of balanol analogues in charge state combination I to PKA and B. The respective correlation coefficients to experimental binding energy over 100 ns of MD simulations. Each data point in A  was obtained from a 10-ns sliding window every 10 ns. Error bars in A  were obtained from $\Delta  {G}_{MMGBSA}^{{}^{\circ}}$ calculations of 100 snapshots within 10 ns trajectory. Error bars in B  were derived from the errors of experimental *K*_d_ values. (PDF 298 kb)

Additional file 2: Figure S2. A. Trajectory of $\Delta  {G}_{MMGBSA}^{{}^{\circ}}$ of balanol analogues in charge state combination **II** to PKA and B. The respective correlation coefficients to experimental binding energy over 100 ns of MD simulations. Each data point in A was obtained from a 10-ns sliding window every 10 ns. Error bars in A were obtained from $\Delta  {G}_{MMGBSA}^{{}^{\circ}}$ calculations of 100 snapshots within 10 ns trajectory. Error bars in B  were derived from the errors of experimental *K*_d_ values. (PDF 255 kb)

Additional file 3: Figure S3.A. Trajectory of $\Delta  {G}_{MMGBSA}^{{}^{\circ}}$ of balanol analogues in charge state combination I to PKCε and B. the respective correlation coefficients to experimental binding energy over 100 ns of MD simulations. Each data point in A was obtained from a 10-ns sliding window every 10 ns. Error bars in A were obtained from $\Delta  {G}_{MMGBSA}^{{}^{\circ}}$ calculations of 100 snapshots within 10 ns trajectory. Error bars in B were derived from the errors of experimental *K*_d_ values. (PDF 258 kb)

## Abbreviations

ATP: Adenosine triphosphate
Balanol: (-)-balanol
MD: Molecular dynamics
MMGBSA: Molecular Mechanics Generalized Born Surface Area
MSEP: Molecular surface electrostatic potential
PKA: Protein kinase A
PKC: Protein kinase C
