# Supplementary material for: Exploration of charge states of balanol analogues acting as ATP-competitive inhibitors in kinases
Source: BMC Bioinformatics. 2017 Dec 28;18(Suppl 16):572. doi: 10.1186/s12859-017-1955-7 (PMC5751415; doi:10.1186/s12859-017-1955-7)
Supplement: Supplementary file 1 — A. Trajectory of $\Delta {G}_{MMGBSA}^{{}^{\circ}}$∆GMMGBSA° of balanol analogues in charge state combination I to PKA and B. The respective correlation coefficients to experimental binding energy over 100 ns of MD simulations. Each data point in A was obtained from a 10-ns sliding window every 10 ns. Error bars in A were obtained from $\Delta {G}_{MMGBSA}^{{}^{\circ}}$∆GMMGBSA° calculations of 100 snapshots within 10 ns trajectory. Error bars in B were derived from the errors of experimental K d values. (PDF 298 kb) [file 12859_2017_1955_MOESM1_ESM.pdf]

Additional file

Supplementary Information

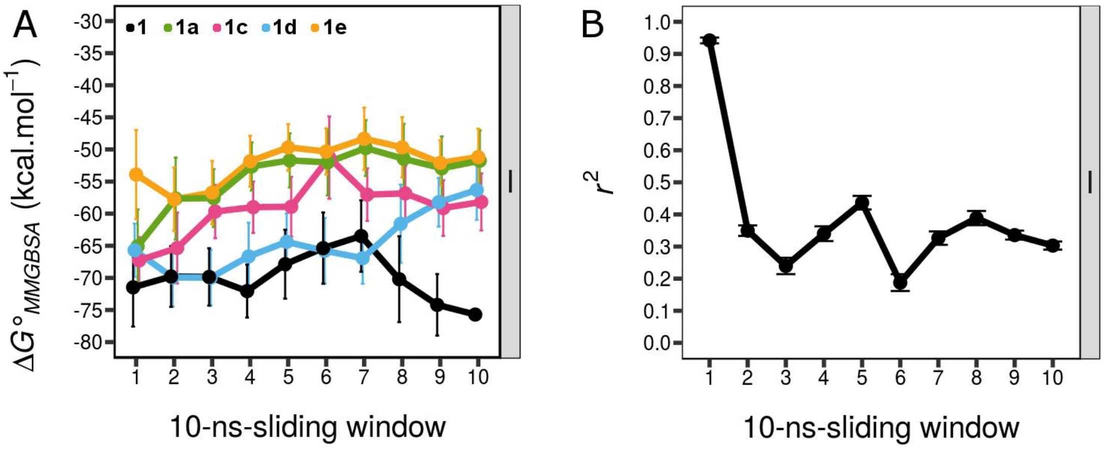

Figure S1 – (A) Trajectory of $\Delta G^{\circ}_{MMGBSA}$ of balanol analogues in charge state combination I to PKA and (B) the respective correlation coefficients to experimental binding energy over 100 ns of MD simulations.

Each data point in (A) was obtained from a 10-ns sliding window every 10 ns. Error bars in (A) were obtained from $\Delta G^{\circ}_{MMGBSA}$ calculations of 100 snapshots within 10 ns trajectory. Error bars in (B) were derived from the errors of experimental K_d values.
