# Supplementary material for: Exploration of charge states of balanol analogues acting as ATP-competitive inhibitors in kinases
Source: BMC Bioinformatics. 2017 Dec 28;18(Suppl 16):572. doi: 10.1186/s12859-017-1955-7 (PMC5751415; doi:10.1186/s12859-017-1955-7)
Supplement: Supplementary file 2 — A. Trajectory of $\Delta {G}_{MMGBSA}^{{}^{\circ}}$∆GMMGBSA° of balanol analogues in charge state combination II to PKA and B. The respective correlation coefficients to experimental binding energy over 100 ns of MD simulations. Each data point in A was obtained from a 10-ns sliding window every 10 ns. Error bars in A were obtained from $\Delta {G}_{MMGBSA}^{{}^{\circ}}$∆GMMGBSA° calculations of 100 snapshots within 10 ns trajectory. Error bars in B were derived from the errors of experimental K d values. (PDF 255 kb) [file 12859_2017_1955_MOESM2_ESM.pdf]

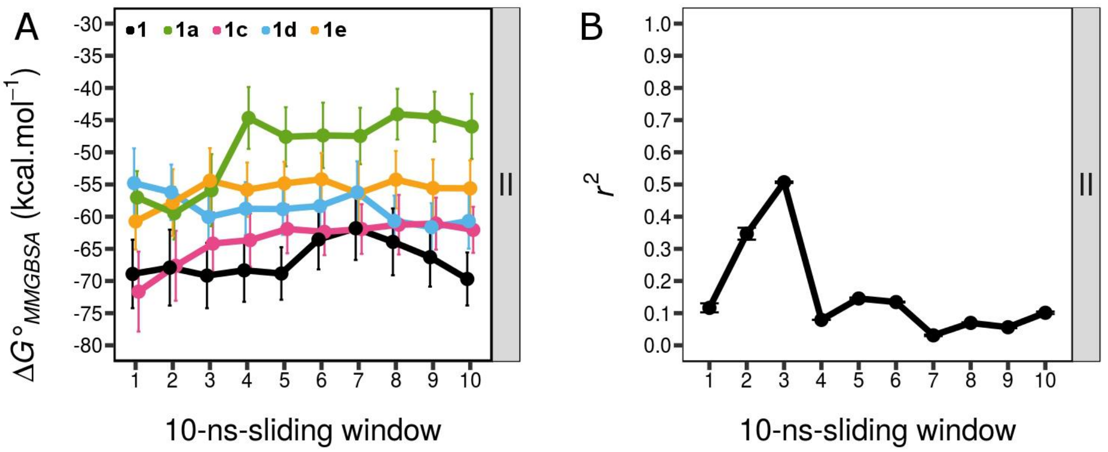

Figure S2 – (A) Trajectory of $\Delta G^{\circ}_{MMGBSA}$ of balanol analogues in charge state combination II to PKA and (B) the respective correlation coefficients to experimental binding energy over 100 ns of MD simulations.

Each data point in (A) was obtained from a 10-ns sliding window every 10 ns. Error bars in (A) were obtained from $\Delta G^{\circ}_{MMGBSA}$ calculations of 100 snapshots within 10 ns trajectory. Error bars in (B) were derived from the errors of experimental K_{d} values.
